# ATBF1 Participates in Dual Functions of TGF-β via Regulation of Gene Expression and Protein Translocalization

**DOI:** 10.3390/biom10050807

**Published:** 2020-05-24

**Authors:** Mei Li, Anqi Zhang, Yanan Zheng, Jiajing Li, Jiyuan Zhao

**Affiliations:** 1 Zhejiang Key Laboratory of Pathophysiology, School of Medicine, Ningbo University, Ningbo 315211, Zhejiang, China; meili@nbu.edu.cn (M.L.); dreamangelzaq@126.com (A.Z.); zhengyn1229@163.com (Y.Z.); lijiajingnbu@163.com (J.L.); 2 Ningbo Institute of Medical Sciences, Ningbo 315016, Zhejiang, China

**Keywords:** TGF-β, ATBF1, epithelial-mesenchymal transition (EMT), cell proliferation, gene expression, translocalization

## Abstract

TGF-β is a critical cytokine to regulate multiple pathophysiological functions. For tumor development and progression, TGF-β was reported to play dual functions as a tumor suppressor and epithelial-mesenchymal transition (EMT) inducer. The mechanism of the TGF-β signaling pathway is essential for TGF-β/Smad-targeted therapy in clinic. Here, ATBF1 was demonstrated to participate in dual functions of TGF-β via different ways. On one hand, ATBF1 expression level was associated with EMT and migration induced by TGF-β. After TGF-β treatment, ATBF1 expression was reduced in a dose- and time-dependent manner, along with the alteration of cell morphology and EMT marker expression. Knockdown of ATBF1 by siRNA further promoted EMT progression and cell migration. On the other hand, ATBF1 localization was associated with cell proliferation inhibited by TGF-β. The number of cells with nucleus localization of ATBF1 in TGF-β activation group was much higher than that in control group. After that, knockdown of ATBF1 by siRNA rescued the inhibition of cell proliferation affected by TGF-β. These data revealed that ATBF1 is a key gene for the dual roles of TGF-β, which may contribute to future therapy.

## 1. Introduction

TGF-β contributes to multiple pathophysiological functions, including cellular homeostasis, angiogenesis, immune response, embryonic development, wound healing, and tissue remodeling [1,2,3,4]. The balance and relations between multipotent cellular effects are essential for TGF-β’s functions [5]. Among these, inhibition of cell proliferation and induction of epithelial-mesenchymal transition (EMT) are two fundamental effects of TGF-β, which contributes to both physiological processes and progression of diseases [6,7].

Recently, the dual roles of TGF-β have been appeared as both a tumor suppressor and a tumor promoter during tumor development and progression, including breast cancer [8], squamous cell carcinoma [9] and gastrointestinal cancers [10]. In premalignant cells, it acted as a tumor suppressor. By contrast, during the progression of breast cancer to malignant phases, TGF-β elicited tumor-promoting effects by driving the EMT, which enhanced tumor cell migration, invasion, and ultimately metastasis to distant organs [11]. Moreover, the growth inhibition affected by TGF-β was also associated with TGF-β-induced EMT in normal cells. The balance in the relationship between growth inhibition and EMT promotion determined the extent of cellular fates [12]. However, the molecular mechanism of the dual effects of TGF-β was not clear. The key genes in the TGF-β signaling pathway were essential for TGF-β/Smad-targeted therapies.

AT-motif binding factor 1 (ATBF1, also named ZFHX3) was originally identified as a transcription factor containing four homeodomains and multiple zinc-finger motifs [13]. Accumulated evidence demonstrated multiple functions of ATBF1 protein, including cellular proliferation and differentiation [14,15], embryonic and tissue development [16,17,18], and cancer progression [19,20].

Previously, ATBF1 was reported as a transcription factor to regulate the transcription of target genes by binding to their promoters [15,21]. In breast epithelial cells, ATBF1 participated in hormone–hormone receptor signaling pathways, including the estrogen–estrogen receptor pathway (E_2_-ER) [14,18,22], the progesterone–progesterone receptor (Pg-PR) pathway [23], and the prolactin–prolactin receptor (Prl-PrlR) pathway [24], which affected breast epithelial cell differentiation, mammary gland development, and breast tumorigenesis. In ER-positive breast cancer cells, ATBF1 was reported to inhibit mRNA transcription of ER target genes by selectively competing with AIB1 for binding to ER, and finally inhibited ER functions [14].

Recently, accumulated evidence has demonstrated that mislocalization of ATBF1 occurred from the nucleus to the cytoplasm in gastric, skin, head and neck, blander, and breast cancers [25,26,27,28,29], which has attracted the attention of researchers. The results of ATBF1 mislocalization to cytoplasm is associated with cell differentiation and histophathologic progression (including cancer metastasis). Moreover, in ER-positive cells, translocation of ATBF1 from cytoplasm to nucleus was associated with mitosis and cell proliferation in an ER-dependent manner [29]. In gastric cancer cells, translocation of ATBF1 from cytoplasm to nucleus was induced by TGF-β signal activation [26]. However, the functions of ATBF1 in TGF-β signaling pathway were not clarified. The effects of TGF-β were highly different due to the high heterogeneity and diversity in cell lineage, cell environment and the properties of cell response to TGF-β stimulation.

HaCaT is a human keratinocytes cell line and a classic EMT model in vitro. In the present study, our aim was to investigate the functions of ATBF1 in the dual effects of TGF-β in HaCaT cells. At first, alteration of ATBF1 expression was examined during EMT progression induced by TGF-β at different concentrations and time points. ATBF1 siRNA was used to knockdown the expression of ATBF1 and determine its function on EMT. The expression of EMT markers and wound healing assay were assessed. Then, translocalization of ATBF1 under TGF-β treatment in normal cells and its function on inhibition of cell growth were investigated.

## 2. Materials and Methods

### 2.1. Reagents, Primers, and siRNAs

The following reagents were purchased from their respective vendors: TGF-β (PeproTech, Inc., Jiangsu, China); anti-ATBF1 primary antibody (Santa Cruz Biotechnology, Shanghai, China); Alexa Fluor^®^ 488 (Cell Signaling Technology (CST), Shanghai, China); 4′,6-diamidino-2-phenylindole (DAPI) (CST); Lipofectamine RNAiMAX (ThermoFisher Scientific, Shanghai, China); Cell Counting Kit (TransGen BiotechCo., Ltd., Beijing, China); One-Step gDNA Removal and cDNA Synthesis Supermix (TransGen Biotech); and TransStart^®^ Top Green qPCR SuperMix (TransGen Biotech).

Primers of ATBF1, E-cad, N-cad, and c-myc were synthesized from Shanghai Generay Biotech Co., Ltd. The sequences of gene primers are listed in Table 1. Small interfering RNAs (siRNAs) were synthesized from Shanghai GenePharma Co., Ltd. The sequence of siATBF1 is 5′-AGAAUAUCCUGCUAGUACA-3′.

### 2.2. Cell Culture and TGF-β Treatment

HaCaT cells were purchased from BeNa Culture Collection (Beijing, China). The cells were cultured in MEMα medium (Gibco, Shanghai, China) supplemented with 15% FBS (Gibco, Australian origin) and 1% pencillin-streptomycin (TransGen Biotech). For EMT assessment, the cells were seeded on tissue culture plates at the confluence of ~80%. On the next day, the cells were starved in MEMα medium with 1% FBS for 24 h, following with TGF-β treatment at desired concentrations or different time points. For immunofluorescence staining and cell proliferation assay, the cell seeding confluence was 30%~40%.

### 2.3. RNA Interference (RNAi)

Cells were seeded on a 24-well plate at the confluence of 30%–40%. The next day, the cells were starved in 500 μL blank medium for 1 h. According to the instructions of Lipofectamine RNAiMAX, control siRNA (siControl) or ATBF1 siRNA (siATBF1) were diluted in 50 μL blank medium with gentle mixture. RNAiMAX reagent (1 μL) was separately diluted in 50 μL blank medium with gentle mixture. The diluted siRNA and lipid reagent were combined together, gently mixed, and incubated for 20 min at room temperature. The mixture was added into each well containing cells. The mixture medium was changed with fresh complete medium after 6 h. The cells were cultured for another two days, following TGF-β treatment.

### 2.4. RNA Extraction, RT-PCR, and Real-Time qPCR

Total mRNA isolation, reverse transcription-polymerase chain reaction (RT-PCR) and real-time quantitative PCR (qPCR) performance were operated as previously reported [30]. Briefly, cells were rinsed with PBS and lysed in RNA-Solv Reagent (R6830-02, Omega, Guangzhou, China) according to the manufacturer’s instructions. The concentration and purity of mRNA were assessed by UV absorbance at 230 nm, 260 nm, and 280 nm using a microplate spectrophotometer. The first-strand cDNA was synthesized using the TransScript One-Step gDNA Removal and cDNA Synthesis SuperMix kit. The relative gene expression on mRNA levels was assessed by RT-PCR and real-time qPCR under comparative Ct method (2^−(ΔΔC(t))^) calculation. GAPDH was introduced as an internal control.

### 2.5. Wound-Healing Assay

Small interfering RNA (siRNA)-transfected HaCaT cells were grown on a six-well tissue culture plate until they reached 100% confluency. The confluent cells were serum-starved in 1% FBS for 24 h. The fully confluent sheet of cells was scratched by a sterile 1000μL tip. Scratched cells were washed twice with serum-starved medium, following with or without TGF-β (10 ng/mL) treatment for up to 48 h. The photos were taken under an optical microscope. Triplicate experiments were performed, and representative images were shown.

### 2.6. Cell Proliferation Assay

The cells were seeded on a 24-well plate and transfected with siControl or siATBF1 on the next day. Two days later, the cells were treated with TGF-β at the concentration of 10 ng/mL. The cell number was determined by Cell Counting Kit (CCK) on days 1 and 3 according to manufacturer’s instructions. Briefly, the cells were cultured with 500 μL fresh medium and 50 μL CCK solution, followed by incubation at 37 °C for 2 h. The medium was transferred into a 96-well plate, and absorbance at 450 nm was examined. Triplicates were performed for each group, and three examinations were assessed for each sample.

### 2.7. Immunofluorescence Staining (IF Staining)

The samples were fixed in 4% formaldehyde for 15 min at room temperature, following with PBS washing. After that, the samples were covered with ice-cold 100% methanol for 10 min at −20 °C and rinsed in PBS. Blocking buffer (5% normal serum/0.3% triton X-100/PBS) were added on the samples and incubated for 60 min at RT. ATBF1 antibody was diluted in 1%BSA/0.3% triton X-100/PBS and incubated on the samples overnight at 4°C. Secondary antibody conjugated with Alexa Fluor^®^ 488 was applied. DAPI was used to show nuclei, and antifade solution was used for sample stocking.

### 2.8. Statistical Analysis

Statistical analyses were performed using SPSS^®^ statistical software (SPSS Inc., Chicago, IL, USA). Student’s *t* test was used to determine statistical differences between two groups, whereas one-way ANOVA or univariate analysis was used to compare three or more groups. *p*Values less than 0.05 were considered statistically significant.

## 3. Results

### 3.1. ATBF1 Expression was Down-Regulated during EMT Progression Induced by TGF-β

**Figure 1:**
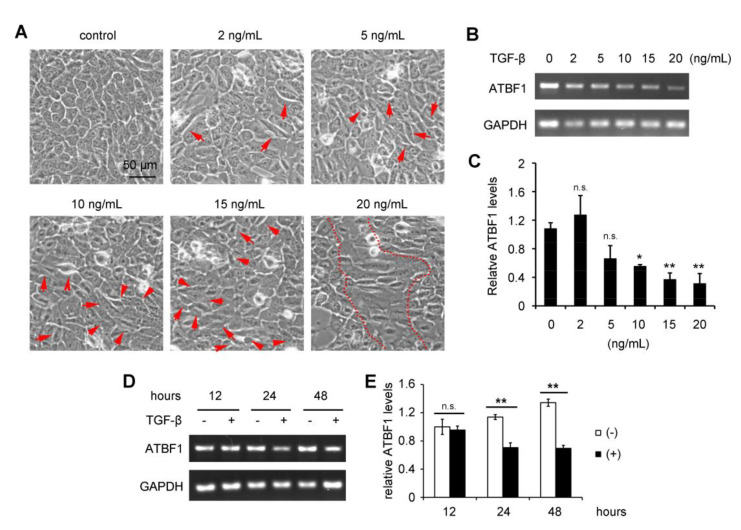
TGF-β reduced ATBF1 expression in a dose- and time-dependent manner, along with epithelial-mesenchymal transition (EMT) induction. HaCaT cell as a classic EMT modelwasintroduced. Cell morphology was imaged under a phase contrast microscope (**A**). Expression of ATBF1 was assessed by RT-PCR after TGF-β treatment at different concentrations (**B**) or different time points (**D**). The relative ATBF1 levels in different groups were quantified by ImageJ software (**C**,**E**). * *p* < 0.01; ** *p* < 0.001; n.s., no significance; compared with control group (no TGF-β treatment). Triplicates were performed for each group.

To test whether ATBF1 takes parts in epithelium-to-mesenchymal transition (EMT) process, we induced EMT in HaCaT cells by TGF-β treatment at gradient concentrations or different time points (Figure 1). Consistently, the morphology of the cells was altered from ovoid to fusiform after TGF-β treatment. More fusiform cells were observed along with higher concentration of TGF-β (Figure 1A). During the EMT progression, ATBF1 expression was down-regulated in a dose- and time-dependent manner (Figure 1B,D). The significant difference occurred at the lowest concentration of 10 ng/mL and the shortest time of 24 h (Figure 1C,E). The results indicated that ATBF1 expression was regulated by TGF-β during EMT progression.

### 3.2. ATBF1 Expression Silencing Enhanced EMT Progression under Activation of TGF-β

**Figure 2:**
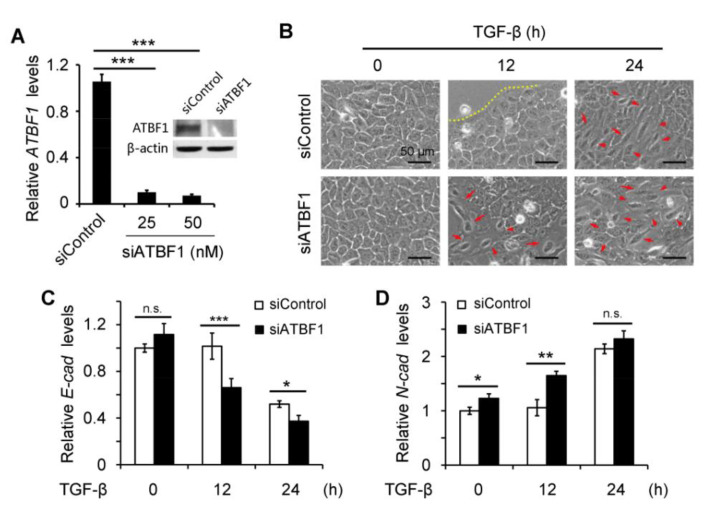
Knockdown of ATBF1 by small interfering RNA (siRNA) promoted EMT progression induced by TGF-β. The efficiency of ATBF1 siRNA was measured by real-time PCR at the concentrations of 25 nM and 50 nM and by western blot at the concentration of 25 nM (**A**). the morphology of cells with or without TGF-β (10 ng/mL) treatment was imaged under a phase contrast microscope (**B**). More mesenchymal-like cells were observed in siATBF1 group after TGF-β treatment for 12 h and 24 h, compared with siControl group (**B**). The expression of E-cad (**C**) and N-cad (**D**) was measured by real-time PCR. *p < 0.05; **p < 0.01; ***p < 0.001; n.s., no significance. Triplicates were performed for each group.

Small interfering RNAs (siRNAs) were used to silence the expression of ATBF1 to investigate the role of ATBF1 on EMT (Figure 2). Efficiency of ATBF1 siRNA was examined by real-time PCR and more than 90% of ATBF1 mRNA was knocked down by 25 nM siATBF1 (Figure 2A). Consistent with mRNA expression, siATBF1 at 25 nM was sufficient to lose the protein expression of ATBF1 (Figure 2A). As shown in Figure 2B, cell morphology was not altered by siATBF1 without TGF-β treatment, along with no alteration of E-cad expression (Figure 2C), although N-cad expression was increased (Figure 2D). However, under the treatment of TGF-β, down-regulation of ATBF1 by siRNA significantly promoted EMT progression. As early as 12 h, fusiform cells were observed in the siATBF1 group, while the cells were still ovoid in the control group (Figure 2B). Meanwhile, the expression of EMT markers was significantly altered by siATBF1 as early as 12 h with down-regulation of E-cad (Figure 2C) and up-regulation of N-cad Figure 2D). The results indicated that loss of ATBF1 promoted EMT progression after activation by TGF-β, but ATBF1 silencing alone was not sufficient to induce EMT.

### 3.3. ATBF1 Expression Silencing Enhanced Cell Migration under Activation of TGF-β

**Figure 3:**
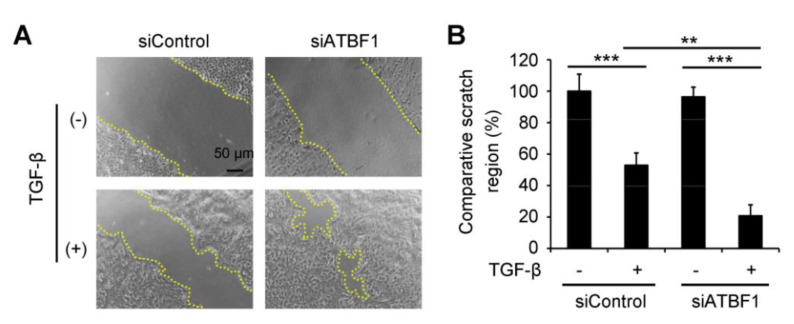
Knockdown of ATBF1 by siRNA promoted cell migration under activation of TGF-β. Wound healing assay was used to assess the cell migration. Cells were transfected with siControl or siATBF1 at 25 nM. (**A**) Morphology of cells with or without TGF-β treatment was imaged under a phase contrast microscope. Yellow dotted line showed the edge of the scratch. (**B**) Scratch region was measured by ImageJ software. Comparative scratch region was defined as the scratch region in each group divided by the scratch region in the control group. ** *p* < 0.01; *** *p* < 0.001. Triplicates were performed for each group.

Wound-healing assay was introduced to investigate cell migration effected by siATBF1 under TGF-β treatment (Figure 3). After siATBF1 transfection, starvation was used to inhibit cell proliferation during wound-healing process. Without TGF-β treatment, the scratch region was comparable between siControl and siATBF1 groups (Figure 3A,B). However, the scratch region was much smaller in the siATBF1 group than that in the siControl group after TGF-β treatment for 48 h (Figure 3A,B), which indicated that the effect of ATBF1 on cell migration was dependent on activation of the TGF-β signaling pathway.

### 3.4. TGF-β Treatment Induced ATBF1 Translocalization from Cytoplasm to Nuclear

**Figure 4:**
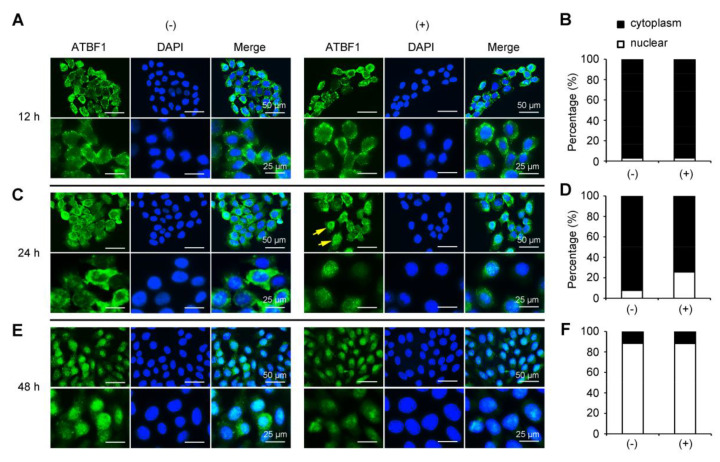
TGF-β promoted ATBF1 translocation from cytoplasm to nucleus. (**A**,**C**,**E**) IF staining for ATBF1 proteins (green) after TGF-β treatment for 12 (A), 24 (C), and 48 h (E). DAPI staining (blue) was used to show cell nucleus. (**B**,**D**,**F**) Cells with different localization of ATBF1 were counted from 10 random images in each group. ATBF1 was mainly localized in cytoplasm at 12 h (A,B) and in nucleus at 48 h (E,F) in both cells with (+) or without (−) TGF-β treatment. However, the cell ratio with nucleus localization of ATBF1 was higher in TGF-β group than that in the control group at 24 h (C,D).

Previous studies have demonstrated that the translocation of ATBF1 is associated with histopathologic progression in head and neck squamous cell carcinoma [27] and is regulated by E_2_-ER signaling pathway [29]. Here, we found that the translocalization of ATBF1 is also induced by TGF-β treatment in normal cells. As shown in Figure 4A, the ATBF1 protein was mainly localized in cytoplasm after starvation and TGF-β treatment for 12 h. The ratio of the cells with ATBF1 cytoplasm localization was more than 97% with or without TGF-β treatment (Figure 4B). Later, after 24 h treatment of TGF-β, translocation of ATBF1 from the cytoplasm to the nucleus occurred in the partial cells (Figure 4C). The ratio of the cells with ATBF1 nucleus localization was higher in the TGF-β treatment group than in the control group (Figure 4D). By 48 h treatment, ATBF1 protein was mainly localized in the nucleus in most of the cells in both groups (with or without TGF-β treatment) (Figure 4E,F). Previous studies have demonstrated that ATBF1 is a tumor suppressor gene that inhibits cell proliferation [14,19]. The function was based on the nucleus localization ATBF1. Thus, translocation of ATBF1 from the cytoplasm to the nucleus induced by TGF-β may contribute to the inhibition of cell proliferation affected by TGF-β.

### 3.5. ATBF1 Expression Silencing Rescued Inhibition of Cell Proliferation Induced by TGF-β

**Figure 5:**
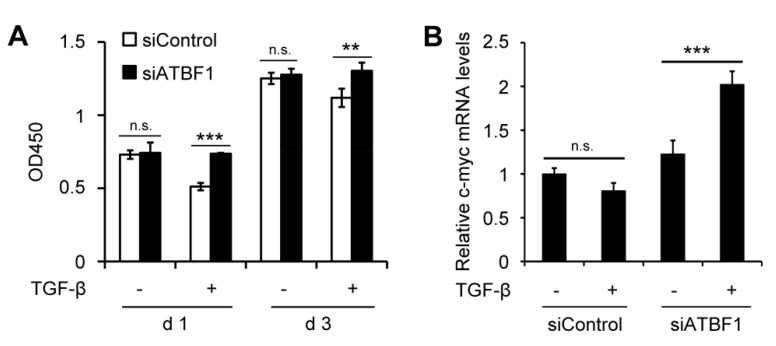
Knockdown of ATBF1 by siRNA rescued the inhibition of cell proliferation induced by TGF-β. (**A**) Cells were transfected with siControl or siATBF1 at 25 nM. Cell counting kit (CCK) was used to assay proliferation of cells with or without TGF-β treatment for one or three days. (**B**) The expression of c-myc (a key gene for cell proliferation) was measured by real-time qPCR. ** *p* < 0.01; *** *p* < 0.001; n.s., no significance. Triplicates were performed for each group.

In HaCaT cells, no significant difference of cell proliferation was observed between siControl and siATBF1 groups without TGF-β treatment on day 1 and day 3 (Figure 5A). However, cell proliferation was significantly inhibited by TGF-β treatment in the siControl group, and the inhibition was rescued by the loss of ATBF1 (siATBF1) (Figure 5A). Molecular assay revealed that c-myc might take parts in the process. Consistent with cell proliferation, the expression of c-myc was reduced by TGF-β treatment in siControl and was increased by siATBF1 under TGF-β treatment (Figure 5B). The results further indicated that the inhibition of ATBF1 on cell proliferation was also dependent on activation of TGF-β signaling pathway, as in the effect on cell migration in HaCaT cells. Thus, ATBF1 was essential for the function of TGF-β.

## 4. Discussion

TGF-β plays dual effects during tumor progression: proliferation inhibition at early stage and EMT induction at late stage [31]. The molecular mechanism of TGF-β signaling pathway is essential for clinical therapy. In the present study, we found that the tumor suppresser gene ATBF1 participated in the TGF-β pathway and regulated the dual effects of TGF-β via different ways. On one hand, TGF-β reduced ATBF1 expression to promote EMT progression. On the other hand, TGF-β induced translocation of ATBF1 from the cytoplasm to the nucleus in order to inhibit cell proliferation.

TGF-β is a pivotal cytokine to activate EMT associated factors, which has been shown to promote tumor migration and invasion. In recent years, several studies have demonstrated that EMT could be initiated by various transcription factors or external signals under activation of TGF-β signaling [32,33,34,35]. These factors or signals have been reported to be associated with tumor progression in clinic. For example, calreticulin (CRT), as an oncogenic protein, was interrelated with TNM staging and lymph node metastasis of nasopharyngeal carcinoma [33]. Further mechanisms study showed that CRT induced EMT, migration, and invasion by the upregulation of neuropilin-1 (NRP1) expression to affect SMAD3 phosphorylation [33]. Moreover, in bladder cancer, nucleolar and spindle associated protein 1 (NUSAP1) was upregulated, and its expression was closely related to the poor prognosis of patients, which was also demonstrated to regulate EMT via the TGF-β signaling pathway, as well as p-Smad2/3 and vimentin expression [34]. In the present study, we focused on a tumor suppression gene ATBF1, and the expression of ATBF1 was demonstrated to be downregulated by TGF-β treatment along with EMT induction in a dose- and time-dependent manner. Although knockdown of ATBF1 by siRNA further promoted the EMT process and cell migration induced by TGF-β, these functions were dependent on the activation of the TGF-β signaling pathway. Without TGF-β, downregulation of ATBF1 alone could not induce EMT, and no effect was found in the wound healing assay compared with the control group.

Besides EMT induction and tumor promotion, TGF-β also plays its role in cell growth. In prostate tissue, TGF-β is active in the regulation of the balance between epithelial cell proliferation and apoptosis through stromal epithelial via the androgen receptor action, which contributes to prostate development and regeneration [36]. During tumor initiation, TGF-β acted as a tumor suppressor and promoted cell cycle arrest and apoptosis to inhibit cell proliferation [10]. However, ATBF1 was also reported as a tumor suppressor gene in multiple cancers to inhibit cell proliferation, which was contradictory with the downregulation of ATBF1 induced by TGF-β. Here, we found TGF-β might inhibit cell proliferation with another mechanism—the translocation of ATBF1 from the cytoplasm to the nucleus. Previously, ATBF1 was identified as a transcription factor with multi zinc fingers to inhibit promoter activities of target genes. Thus, nucleus localization of ATBF1 was critical for its function in proliferation inhibition. In breast epithelial cells, we demonstrated that the localization of ATBF1 on the chromosome was associated with the whole mitosis process, including prophase, metaphase, anaphase, and telophase [29]. In gastric cancer cells, endogenous ATBF1 was translocated to the nucleus from cytoplasm under treatment of TGF-β [26]. Consistently, more cells with nucleus ATBF1were detected after TGF-β treatment for 24 h in HaCaT cells, while almost all cells were detected with ATBF1 nucleus after 48 h, even in the control group. Therefore, earlier induction of ATBF1 translocation might be important for its function regarding the inhibition of cell proliferation by TGF-β. The balance of ATBF1 translocation and expression decided the cell fate.

## 5. Conclusions

As a tumor suppressor gene, downregulation and translocalization of ATBF1 frequently occurs in multiple cancers. In the present study, we clarified the potential functions of ATBF1 in TGF-β signaling pathways via different mechanisms. TGF-β reduced ATBF1 expression during EMT progression, and loss of ATBF1 by siRNA further promoted EMT and cell migration induced by TGF-β. Then, translocation of ATBF1 from the cytoplasm to the nucleus inhibited cell proliferation under TGF-β treatment. Thus, ATBF1 may be a potential target gene with dual effects in TGF-β signaling pathway for clinical therapy.

## Figures and Tables

**Table 1:** Primer sequences for PCR amplification.

Gene		Sequence	Length (bps)
ATBF1 (RT-PCR)	Forward	CATCAAGGAGGGCGGCAA	300
	Reverse	CTCTCGCTTCGCTGGTGCTT	
ATBF1 (real-time qPCR)	Forward	TGTTCCAGATCGAGATGGGAAT	75
	Reverse	CTTTCCCAGATCCTCTGAGGTTT	
E-cad	Forward	TGAAGGTGACAGAGCCTCTGGAT	151
	Reverse	TGGGTGAATTCGGGCTTGTT	
N-Cad	Forward	GACAATGCCCCTCAAGTGTT	179
	Reverse	CCATTAAGCCGAGTGATGGT	
c-myc	Forward	TCAAGAGGTGCCACGTCTCC	80
	Reverse	TCTTGGCAGCAGGATAGTCCTT	
GAPDH	Forward	GGTGGTCTCCTCTGACTTCAACA	127
	Reverse	GTTGCTGTAGCCAAATTCGTTGT	
